# Control of Rabies as a Victim of Its Own Success: Perception of Risk within a Latin American Population

**DOI:** 10.4269/ajtmh.20-0728

**Published:** 2020-07-13

**Authors:** Felipe Rocha

**Affiliations:** Pan American Center for Foot-and-Mouth Disease and Veterinary Public Health, Pan American Health Organization/World Health Organization (PANAFTOSA-PAHOWHO)

Of the zoonotic diseases that afflict man, rabies is among the most well-known, having been described for at least four millennia.^1^ Historically, in the urban environment, the dog is responsible for the maintenance of the disease and is its main source of transmission to man. During the 1980s, Latin America was affected with hundreds of human cases of rabies.^2^ Although there is no cure, timely care through immunization of people exposed to rabies is one of the key tools for its prevention. This measure, combined with vaccination campaigns of the canine population, sensitive and active epidemiological surveillance, and awareness and education of the population, forms the basis of disease control and elimination programs in the region.^3^ In addition, the population’s knowledge and perception of the disease is another determining factor for disease control. As a result, Latin America has achieved a reduction of 95% in the incidence of human rabies. Gradually, like other diseases that have been successfully eliminated, rabies has become less relevant and lately neglected.^4^ After an exposure, an individual must seek primary health care to receive guidance on rabies, to have a wound treated, and to receive postexposure immunization, which prevents the development of the disease. Therefore, because it is a disease with straightforward, effective prevention, rabies control is seen as a parameter to measure the quality of health services.

In a study published in this issue of the *American Journal of Tropical Medicine and Hygiene*, De la Puente-León and others^5^ studied the population of Arequipa, Peru, one of the pockets of rabies reintroduction in dogs. Through interviews in urban and peri-urban areas of the city, which have very different economic and social arrangements, the study sought to quantify the extent of dog bites and to characterize factors linked to healthcare-seeking behaviors. The results revealed a higher rate of dog bites in the peripheral areas of the city, areas that are often more needy and in which potentially rabies-exposed individuals were less likely to seek basic health care.

Accessibility of health services is one of the crucial and determining factors driving the seeking of care after a potential rabies exposure. Other studies have discussed the challenge of accessing vaccination sites during canine vaccination campaigns, with proposals and models to identify the best distribution to increase accessibility.^6^ In this study, the authors highlighted differences between urban and peri-urban neighborhoods, identifying distances of up to 2.5 km between residences and health facilities in peri-urban neighborhoods in comparison to an average distance of 450 m in urban neighborhoods. This disparity proved to be decisive, with almost twice as many searches for care in the urban area compared with the peri-urban area.

The search for health care does not depend solely on accessibility of healthcare centers. It also depends on other factors over which governments have no direct control, such as the population’s perception of rabies risk and the interest in seeking medical assistance in the event of a possible exposure. With progress in the control of the disease, little by little, rabies is losing attention from the population, with few occurrences across the continent to warn those who neglect it.^3^ The direct consequence is observed in this study: a low demand for postexposure rabies prophylaxis. Thus, rabies control ends up a victim of its own success: advances in disease control lead to a significant decrease in incidence, which leads to a decrease in attention amid so many other more prevalent diseases, which in turn leads to relaxation of preventive measures and actions. The result is the maintenance of the disease at a low, but persistent, level, still circulating among the canine population, until a human case occurs, and control and prevention measures are reinforced, increasing awareness among the population.

Another important issue addressed is the extent of potential exposure to the rabies virus. In this sense, two crucial points are studied and discussed, which are the higher rate of dog bites in the peri-urban environment and how epidemiological surveillance should look more at this phenomenon. Among those who work with rabies, it is said that when a case occurs, we are facing a lost battle. This is because the infectious event that culminated in the occurrence of the disease occurred weeks before. Therefore, health systems should pay more attention to the transmission of the disease via dog bite. The population should be more aware of the need to report dog bites and to seek timely care when potential exposures happen. The number of dog bites could serve as indicators to measure variations in the canine population size, to determine the amount of immunobiologicals needed for postexposure prophylaxis, and to assess the level of rabies awareness in the population. In peri-urban areas specifically, a higher rate of bites was observed than urban areas, and as pointed out in the study, there may be many reasons for this discrepancy. In particular, the peri-urban environment provides better conditions for maintaining a large dog population that is neglected by people, thus allowing more infectious contacts between dogs and people, and thus an enabling environment for the rabies virus to survive and continue circulating.

Cases of rabies in humans are not reported in Arequipa, indicating some success in measures to prevent the disease, despite various difficulties in primary health care. However, with rabies established among the canine population, failures in primary health care could represent a great risk to humans. This is why massive dog vaccination campaigns are an important measure, and local authorities should strengthen this pillar of the rabies control program. The study presented in this issue of the *Journal* discusses crucial points that should be considered for the final journey of the Americas toward the control and elimination of human rabies mediated by dogs. The observed findings were for a specific population, but they can serve as indicators for the population of all Latin America. Furthermore, the study present key points beyond rabies, and relevant in general for public health in urban and peri-urban areas of Latin America.
